# Assessing the Incremental Contribution of Common Genomic Variants to Melanoma Risk Prediction in Two Population-Based Studies

**DOI:** 10.1016/j.jid.2018.05.023

**Published:** 2018-12

**Authors:** Anne E. Cust, Martin Drummond, Peter A. Kanetsky, Australian Melanoma Family Study Investigators, Graham J. Mann, Anne E. Cust, Helen Schmid, John L. Hopper, Joanne F. Aitken, Bruce K. Armstrong, Graham G. Giles, Elizabeth Holland, Richard F. Kefford, Mark A. Jenkins, Leeds Case-Control Study Investigators, Julia A. Newton Bishop, Paul Affleck, Jennifer H. Barrett, D. Timothy Bishop, Jane Harrison, Mark M. Iles, Juliette Randerson-Moor, Mark Harland, John C. Taylor, Linda Whittaker, Kairen Kukalizch, Susan Leake, Birute Karpavicius, Sue Haynes, Tricia Mack, May Chan, Yvonne Taylor, John Davies, Paul King, Alisa M. Goldstein, Jennifer H. Barrett, Stuart MacGregor, Matthew H. Law, Mark M. Iles, Minh Bui, John L. Hopper, Myriam Brossard, Florence Demenais, John C. Taylor, Clive Hoggart, Kevin M. Brown, Maria Teresa Landi, Julia A. Newton-Bishop, Graham J. Mann, D. Timothy Bishop

**Affiliations:** 1 Cancer Epidemiology and Prevention Research, Sydney School of Public Health, The University of Sydney, Sydney, Australia; 2 Melanoma Institute Australia, The University of Sydney, Sydney, Australia; 3 Department of Cancer Epidemiology, Moffitt Cancer Center and Research Institute, Tampa, Florida, USA; 4 Human Genetics Program, Division of Cancer Epidemiology and Genetics, National Cancer Institute, Bethesda, Maryland, USA; 5 Section of Epidemiology and Biostatistics, Leeds Institute of Cancer and Pathology, University of Leeds, Leeds, UK; 6 Statistical Genetics Lab, QIMR Berghofer Medical Research Institute, Brisbane, Australia; 7 Centre for Epidemiology and Biostatistics, Melbourne School of Population Health, University of Melbourne, Australia; 8 INSERM, UMR 946, Genetic Variation and Human Diseases Unit, Paris, France; 9 Institut Universitaire d’Hématologie, Université Paris Diderot, Sorbonne Paris Cité, Paris, France; 10 Section of Paediatrics, Division of Infectious Diseases, Department of Medicine, Imperial College London, London, UK; 11 Centre for Cancer Research, Westmead Institute for Medical Research, The University of Sydney, Sydney, Australia

**Keywords:** AUC, area under receiver operating characteristic curves, CI, confidence interval, NRI, net reclassification improvement, OR, odds ratio, SNP, single nucleotide polymorphism

## Abstract

It is unclear to what degree genomic and traditional (phenotypic and environmental) risk factors overlap in their prediction of melanoma risk. We evaluated the incremental contribution of common genomic variants (in pigmentation, nevus, and other pathways) and their overlap with traditional risk factors, using data from two population-based case-control studies from Australia (n = 1,035) and the United Kingdom (n = 1,460) that used the same questionnaires. Polygenic risk scores were derived from 21 gene regions associated with melanoma and odds ratios from published meta-analyses. Logistic regression models were adjusted for age, sex, center, and ancestry. Adding the polygenic risk score to a model with traditional risk factors increased the area under the receiver operating characteristic curve (AUC) by 2.3% (*P* = 0.003) for Australia and by 2.8% (*P* = 0.002) for Leeds. Gene variants in the pigmentation pathway, particularly *MC1R*, were responsible for most of the incremental improvement. In a cross-tabulation of polygenic by traditional tertile risk scores, 59% (Australia) and 49% (Leeds) of participants were categorized in the same (concordant) tertile. Of participants with low traditional risk, 9% (Australia) and 21% (Leeds) had high polygenic risk. Testing of genomic variants can identify people who are susceptible to melanoma despite not having a traditional phenotypic risk profile.

## Introduction

Primary and secondary prevention strategies that reduce sun exposure and encourage increased sun protection and skin examination behaviors are important for reducing melanoma incidence and mortality, particularly for those identified as being at high risk (Aitken et al., 2010, Armstrong and Kricker, 1993, Breitbart et al., 2012, Glanz et al., 2015). To date, identifying people at high risk for melanoma has focused on factors such as family history (Olsen et al., 2010b), phenotypic characteristics (fair skin, skin that burns easily, red hair, moles and freckling (Gandini et al., 2005a), solar and artificial UV radiation exposure (Cust et al., 2011a, Gandini et al., 2005b), and previous keratinocyte skin cancers (Gandini et al., 2005b).

Common genomic variants may help identify people at high risk for melanoma, particularly those who lack these traditional risk factors (Berwick et al., 2014, Cust et al., 2012, Kanetsky et al., 2010). It is becoming more feasible to incorporate genomic profiles into risk prediction tools, given increased understanding of genomic risk factors and increased genomic testing in clinical practice. Development and assessment of melanoma risk prediction models that combine traditional and genomic risk factors is warranted to help improve melanoma prevention and population screening. For example, Australian general practice guidelines state that clinical assessment of melanoma risk should take into account multiple risk factors yet highlight the fact that there are no sufficiently well-validated risk models to assess the combined effects of all risk factors (Royal Australian College of General Practitioners, 2016). Accurate identification of people at high risk for melanoma will also facilitate research on targeted screening strategies for those at higher risk (US Preventive Services Task Force et al., 2016).

Few studies have examined the contribution of multiple genomic risk factors to risk prediction over and above that of traditional risk factors (Fang et al., 2013, Kypreou et al., 2016, Stefanaki et al., 2013). Limitations of previous studies have included lack of external validation, small sample size (Stefanaki et al., 2013), lack of data on nevus phenotype or family history (Fang et al., 2013, Kypreou et al., 2016, Stefanaki et al., 2013), and possible confounding by ethnicity (Fang et al., 2013).

We aimed to evaluate the incremental contribution of common genomic variants to melanoma risk prediction, including the contribution of variants associated with identified biological pathways, using data from two population-based studies in geographically disparate but genetically similar populations (Australia and the UK). Both studies were developed by the GenoMEL (www.genomel.org) melanoma genetics consortium and used the same measurement protocols, facilitating external validation.

## Results

### Participant characteristics

Demographic characteristics of participants in the Australian and Leeds, UK, case-control studies in this analysis are shown in Table 1. The studies had a similar proportion (60%) of female participants but a slightly different mix of European ethnicities; results were similar if restricted to English ethnic background. Participants were younger in the Australian study because it restricted recruitment to onset before age 40 years.

**Table 1:** Characteristics of case and control individuals in the Australian Melanoma Family Study and Leeds case-control study

Characteristic	Australia, n (%)	Leeds, n (%)
Total, case and control individuals	1,035	1,460
Case individuals	578 (56)	964 (66)
Control individuals	457 (44)	496 (34)
Sex		
Female	623 (60)	874 (60)
Male	412 (40)	586 (40)
Age at diagnosis/interview, years^1^		
18–29	268 (26)	61 (4)
30–39	682 (66)	198 (14)
40–49	85 (8)	264 (18)
≥50–69	0 (0)	937 (64)
Ethnic background^2^		
English	649 (63)	1,358 (93)
Scottish, Irish, Welsh	52 (5)	67 (5)
Other Northern European	46 (4)	6 (0)
Southern European	11 (1)	6 (0)
Eastern European	238 (23)	3 (0)
Mixed/other European	39 (4)	20 (1)

1 Leeds case and control individuals were unselected for age at diagnosis. In Australia, all case individuals were younger than 40 years at diagnosis, and all population control individuals were younger than 40 years when ascertained. Case and control individuals could be up to age 44 years at interview for this analysis.

2 Self-reported.

### Association of polygenic risk score with melanoma risk

For each country, there was a 3-fold higher risk of melanoma for participants in the highest versus lowest tertile of polygenic risk score and a 6-fold higher risk in the highest versus lowest decile; the odds ratio (OR) per adjusted standard deviation increase in polygenic score (i.e., OPERA) was 1.75 for Australia and 1.63 for Leeds (Table 2). When evaluated by biological pathway (see Supplementary Table S1 online) the ORs for the pigmentation pathway were similar to the overall polygenic risk score, but the ORs were lower for the telomere/senescence/other pathway (about 50% increased risk for the highest vs. lowest tertile) and the nevus pathway (about 25% nonsignificantly increased risk for the highest vs. lowest tertile).

**Table 2:** Association of an overall polygenic risk score with melanoma risk in the Australia and Leeds studies

Overall Polygenic Risk Score	Australia				Leeds			
	Range	Case Individuals, n (%)	Control Individuals, n (%)	Odds Ratio^1^ (95% CI)	Range	Case Individuals, n (%)	Control Individuals, n (%)	Odds Ratio^1^ (95% CI)
Tertiles								
1	–2.11 to 0.16	103 (18)	152 (33)	1.00	–1.46 to 0.16	160 (17)	163 (33)	1.00
2	0.16 to 0.60	158 (27)	170 (37)	1.38 (0.97–1.94)	0.16 to 0.60	312 (32)	154 (31)	2.09 (1.56–2.82)
3	0.60 to 2.52	317 (55)	135 (30)	3.22 (2.30–4.51)	0.60 to 2.69	492 (51)	179 (36)	2.84 (2.14–3.77)
*P* trend				<0.0001				<0.0001
*P* int^2^								0.29
Deciles								
1	–2.11 to –0.26	24 (4)	43 (9)	1.00	–1.46 to –0.27	48 (5)	53 (11)	1.00
2	–0.26 to –0.05	23 (4)	45 (10)	0.93 (0.45–1.94)	–0.26 to –0.05	38 (4)	50 (10)	0.88 (0.49–1.58)
3	–0.05 to 0.13	37 (6)	43 (9)	1.47 (0.73–2.94)	–0.05 to 0.13	64 (7)	52 (10)	1.45 (0.84–2.50)
4	0.13 to 0.27	51 (9)	54 (12)	1.83 (0.95–3.54)	0.13 to 0.27	83 (9)	42 (8)	2.36 (1.36–4.10)
5	0.28 to 0.37	54 (9)	76 (17)	1.23 (0.65–2.32)	0.27 to 0.37	57 (6)	41 (8)	1.61 (0.91–2.84)
6	0.37 to 0.49	41 (7)	30 (7)	2.51 (1.23–5.13)	0.37 to 0.49	109 (11)	43 (9)	2.99 (1.75–5.10)
7	0.49 to 0.63	49 (8)	41 (9)	2.13 (1.08–4.18)	0.49 to 0.63	91 (9)	55 (11)	1.94 (1.14–3.27)
8	0.64 to 0.82	77 (13)	44 (10)	3.06 (1.60–5.83)	0.64 to 0.83	106 (11)	51 (10)	2.44 (1.45–4.12)
9	0.83 to 1.06	73 (13)	38 (8)	3.03 (1.56–5.88)	0.83 to 1.06	118 (12)	57 (11)	2.44 (1.46–4.08)
10	1.06 to 2.52	149 (26)	43 (9)	5.88 (3.14–11.03)	1.06 to 2.69	250 (26)	52 (10)	5.62 (3.41–9.29)
*P* trend				<0.0001				<0.0001
*P* int^2^								0.28
OPERA^3^				1.75 (1.53–2.01)				1.63 (1.46–1.83)
*P* int^2^								0.40

1 Models are adjusted for demographic and study design factors of age, sex, city of recruitment, and European ancestry.

2 *P*-value for interaction comparing trends across countries.

3 Odds ratio per adjusted standard deviation, stratified by location (Australia/Leeds) and adjusted for age and sex, using the OPERA method (Hopper, 2015).

### Incremental contribution of polygenic risk score based on published risk estimates

Adding the polygenic risk score based on published ORs to a model with traditional risk factors increased the AUC by 2.3% (*P* = 0.003) for Australia and by 2.8% (*P* = 0.002) for Leeds (Table 3). The net reclassification improvement (NRI) was 0.42 (95% confidence interval [CI] = 0.30–0.54) for Australia and 0.29 (95% CI = 0.18–0.39) for Leeds; this was driven more by improvements in specificity (i.e., net movement of control individuals to a lower risk: 29% in Australia and 19% in Leeds) than in sensitivity (net movement of case individuals to a higher risk: 13% in Australia, 9% in Leeds). Single nucleotide polymorphisms (SNPs) in the pigmentation pathway, particularly *MC1R*, were responsible for most of the incremental improvement. Conversely, SNPs in the nevus and other pathways did not significantly improve risk prediction. Most models were well calibrated except for the addition of the nevus pathway SNPs in Australia.

**Table 3:** Incremental contribution of genetic risk factors to risk prediction of melanoma when added to a traditional risk factor model, based on published risk estimates

Risk Factor Model	AUC (95% CI)	Change in AUC From Base Model	*P*-Value^1^	Hosmer- Lemeshow *P*-Value	Improvement in Sensitivity NRI (95% CI)^2^	Improvement in Specificity NRI (95% CI)^2^	Overall Improvement in Classification NRI (95% CI)^2^
Australia (N = 1,035)							
Base model with traditional risk factors^3^	0.72 (0.69 to 0.75)			0.13			
+ *MC1R*	0.73 (0.70 to 0.76)	0.011	0.05	0.47	–0.02 (–0.10 to 0.06)	0.35 (0.26 to 0.43)	0.33 (0.21 to 0.45)
+ Pigmentation pathway	0.74 (0.71 to 0.77)	0.019	0.008	0.58	0.11 (0.03 to 0.19)	0.26 (0.18 to 0.35)	0.38 (0.26 to 0.50)
+ Nevus pathway	0.72 (0.69 to 0.75)	0.001	0.54	<0.01	–0.02 (–0.11 to 0.06)	0.07 (–0.02 to 0.16)	0.04 (–0.08 to 0.17)
+ Telomere, senescence, and other pathway	0.72 (0.69 to 0.75)	0.002	0.36	0.14	–0.06 (–0.14 to 0.02)	0.16 (0.07 to 0.25)	0.10 (–0.02 to 0.22)
+ All SNPs^4^	0.74 (0.71 to 0.77)	0.023	0.003	0.23	0.13 (0.05 to 0.21)	0.29 (0.20 to 0.38)	0.42 (0.30 to 0.54)
Leeds (N = 1,460)							
Base model with traditional risk factors^3^	0.65 (0.62 to 0.68)			0.70			
+ *MC1R*	0.67 (0.64 to 0.70)	0.014	0.02	0.34	–0.10 (–0.16 to –0.03)	0.27 (0.18 to 0.35)	0.17 (0.07 to 0.28)
+ Pigmentation pathway	0.68 (0.66 to 0.71)	0.031	0.0005	0.76	0.09 (0.02 to 0.15)	0.22 (0.13 to 0.30)	0.30 (0.20 to 0.41)
+ Nevus pathway	0.65 (0.62 to 0.68)	0.000	0.98	0.81	–0.03 (–0.10 to 0.03)	0.06 (–0.03 to 0.14)	0.02 (–0.08 to 0.13)
+ Telomere, senescence, and other pathway	0.66 (0.63 to 0.69)	0.004	0.33	0.19	–0.01 (–0.07 to 0.06)	0.10 (0.02 to 0.19)	0.10 (–0.01 to 0.21)
+ All SNPs^4^	0.68 (0.65 to 0.71)	0.028	0.002	0.10	0.09 (0.03 to 0.16)	0.19 (0.11 to 0.28)	0.29 (0.18 to 0.39)

Abbreviations: AUC, area under the receiver operating characteristic curve; CI, confidence interval; NRI, net reclassification improvement; SNP, single nucleotide polymorphism.

1 Chi-square *P*-value for the difference in the AUC when compared with the base model.

2 Based on continuous NRI. Improvement in sensitivity is calculated from reclassification of case individuals improvement in specificity is calculated from reclassification of control individuals, and overall improvement combines the improvements in sensitivity and specificity.

3 Traditional factors include hair color, skin color, eye color, freckling as an adult, skin photosensitivity, self-reported nevi, sunbed use, keratinocyte cancer personal history, first degree family history of melanoma, vacation sun exposure, and blistering sunburns as a child, as well as the demographic and study design factors of age, sex, city of recruitment, and European ancestry.

4 Added as a polygenic risk score, comprising 45 SNPs in 21 genes. The SNPs in each pathway can overlap; the pigmentation pathway includes 14 genes (31 SNPs); nevus pathway includes 7 genes (13 SNPs); and telomere, senescence, and other pathways includes 5 genes (9 SNPs).

### Secondary analyses

Polygenic risk score and traditional risk factors had similar discrimination when compared side by side in separate models (respectively, 0.71 vs. 0.72 for Australia and 0.64 vs. 0.65 for Leeds). The incremental contribution of the polygenic risk score, including pathway-specific scores, was stronger when the models were based on risk estimates derived from the study datasets (AUC increased by 6.0% for Australia and 5.6% for Leeds), but after 10-fold cross-validation, the AUC increase was 2.0% and 1.2%, respectively (see Supplementary Table S2 online).

### Cross-tabulation of polygenic risk score with traditional risk score

In a cross-tabulation of polygenic versus traditional risk scores categorized as tertiles (low, average, high), 59% (Australia) and 49% (Leeds) of participants were categorized in the same (concordant) tertile (Table 4). Of participants with low traditional risk, 9% in Australia and 21% in Leeds had a high polygenic risk, indicating genetic susceptibility despite lack of phenotypic risk features. Conversely, 8% of participants in Australia and 18% in Leeds had a high traditional risk but a low polygenic risk.

**Table 4:** Cross-tabulation of polygenic risk score versus traditional risk score categorized in tertiles^1^

Traditional Risk Score	Polygenic Risk Score, n (%)			
	Tertile 1 (Lower Risk)	Tertile 2 (Average Risk)	Tertile 3 (Higher Risk)	Total
Australia				
Tertile 1 (lower risk)	223 (65)	91 (26)	30 (9)	344 (100)
Tertile 2 (average risk)	94 (27)	160 (46)	91 (26)	345 (100)
Tertile 3 (higher risk)	27 (8)	94 (27)	225 (65)	346 (100)
Total	344	345	346	1,035
Leeds				
Tertile 1 (lower risk)	244 (50)	138 (28)	104 (21)	486 (100)
Tertile 2 (average risk)	153 (31)	209 (43)	125 (26)	487 (100)
Tertile 3 (higher risk)	89 (18)	140 (29)	258 (53)	487 (100)
Total	486	487	487	1,460

1 Both models are adjusted for demographic and study design factors: age, sex, city of recruitment, and European ancestry.

### Parsimonious risk prediction models combining traditional and genomic risk factors

Table 5 lists traditional and genomic risk factors selected as a more parsimonious model for each dataset. The same genetic variants and traditional risk factors were assessed for inclusion in both models, but only those with *P* < 0.20 in the multivariable model were retained. Traditional risk factors contributing to the final models for Australia and Leeds included family history of melanoma, nonmelanoma skin cancer, hair color, and nevus density. Other traditional UV and pigmentation variables retained in the final models differed between countries. A similar number of SNPs was selected for each model (19 for Australia, 18 for Leeds), but only seven overlapped. The AUCs for the Australian model were 0.77 (95% CI = 0.74–0.80) on internal validation and 0.72 (95% CI = 0.69–0.75) on external validation; for the Leeds model the AUCs were 0.72 (95% CI = 0.69–0.75) on internal validation and 0.77 (95% CI = 0.74–0.80) on external validation.

**Table 5:** Development of a risk prediction model for each dataset using model selection^1^

Variable Selected	Australian Model, Odds Ratio (95% CI)^2^	Leeds Model, Odds Ratio (95% CI)^2^
Traditional risk factors		
Family history of melanoma		
None	1.00	1.00
1 or more relative	1.61 (1.05–2.48)	3.38 (1.33–8.59)
Hair color		
Dark brown/black	1.00	1.00
Light brown	1.01 (0.71–1.44)	1.15 (0.79–1.68)
Fair or blonde	1.82 (1.16–2.86)	2.13 (1.32–3.42)
Red	2.76 (1.36–5.60)	1.86 (0.96–3.58)
Nevus density		
None	1.00	1.00
Few	1.19 (0.57–2.48)	1.84 (1.09–3.11)
Some	3.13 (1.50–6.52)	3.93 (2.27–6.79)
Many	5.36 (2.43–11.83)	4.64 (2.36–9.15)
Nonmelanoma skin cancer		
No	1.00	1.00
Yes	2.28 (1.19–4.37)	3.86 (0.77–19.40)
Blistering sunburn as a child		
None	1.00	—
1 or more episodes	0.80 (0.58–1.11)	—
Sunbed use		
None	1.00	—
1–10 sessions	0.96 (0.61–1.51)	—
>10 sessions	1.79 (1.01–3.20)	—
Freckling as an adult		
None/very few	—	1.00
Few/some/many	—	0.73 (0.52–1.02)
Eye color		
Brown or black	—	1.00
Green or hazel	—	1.05 (0.68–1.63)
Blue or grey	—	1.39 (0.89–2.16)
Sun exposure hours on weekends and vacations		
Quartile 1 (lower exposure)		1.00
Quartile 2	—	0.52 (0.34–0.81)
Quartile 3	—	0.61 (0.39–0.96)
Quartile 4 (higher exposure)	—	0.44 (0.27–0.72)
Genomic variants		
rs7412746 (*ARNT*)	0.85 (0.67–1.06)	0.81 (0.65–1.02)
rs62211989 (*ASIP*)	1.90 (1.33–2.71)	1.91 (1.28–2.84)
R151C (*MC1R*)	2.59 (1.77–3.79)	2.75 (1.83–4.13)
R160W (*MC1R*)	1.47 (1.00–2.16)	1.70 (1.15–2.51)
rs2487999 (*OBFC1*)	1.40 (0.95–2.06)	1.37 (0.93–2.01)
rs132985 (*PLA2G6*)	1.19 (0.95–1.48)	0.85 (0.68–1.06)
rs1393350 (*TYR*)	1.32 (1.03–1.70)	1.20 (0.95–1.52)
rs6949072 (*AGR3*)	1.28 (0.94–1.74)	—
rs7274597 (*ASIP*)	0.50 (0.31–0.81)	—
rs76699054 (*CCND1*)	1.40 (0.93–2.12)	—
rs12527588 (*CDKAL1*)	1.56 (0.95–2.55)	—
rs3731217 (*CDKN2A*)	0.79 (0.57–1.09)	—
D84E (*MC1R*)	2.18 (1.02–4.67)	—
I155T (*MC1R*)	2.60 (1.09–6.18)	—
V60L (*MC1R*)	1.74 (1.21–2.50)	—
V92M (*MC1R*)	1.70 (1.17–2.49)	—
rs45430 (*MX2*)	0.72 (0.57–0.90)	—
rs3219090 (PARP1)	0.73 (0.58–0.93)	—
rs2736100 (*TERT*)	0.74 (0.59–0.94)	—
rs34585474 (*AGR3*)	—	1.27 (0.89–1.83)
rs7781130 (*AGR3*)	—	1.59 (0.85–2.96)
rs1801516 (*ATM*)	—	0.77 (0.55–1.07)
rs700635 (*CASP8*)	—	1.27 (0.99–1.63)
rs7776158 (*CDKAL1*)	—	1.36 (1.06–1.75)
rs16953002 (*FTO*)	—	1.27 (0.93–1.73)
R163Q (*MC1R*)	—	0.62 (0.36–1.09)
D294H (*MC1R*)	—	4.11 (1.62–10.46)
rs6517661 (*MX2*)	—	0.75 (0.53–1.07)
rs113908778 (*RAD23B*)	—	0.55 (0.24–1.24)
rs4436178 (*RAD23B*)	—	1.87 (0.82–4.26)

Abbreviation: CI, confidence interval.

1 A risk prediction model was developed separately for each dataset using a backward selection process in which traditional and genomic risk factors with *P* < 0.20 were retained in the multivariable model in addition to forced variables age, sex, city of recruitment, and European ancestry. The same genetic variants and traditional risk factors were assessed for inclusion in both models.

2 Odds ratios derived from the respective dataset, adjusted for all other variables in the model. For genomic variants, the per-allele odds ratio is presented. Values left blank indicate that the factor was not included in the final model for that dataset (Australia/Leeds). The areas under the curve for the Australian model were 0.80 (95% CI = 0.77–0.83) from the development model, 0.77 (95% CI = 0.74–0.80) from internal validation (10-fold cross-validation), and 0.72 (95% CI = 0.69–0.75) from external validation using the Leeds dataset. The areas under the curve for the Leeds model were 0.77 (95% CI = 0.73–0.80) from the development model, 0.72 (95% CI = 0.69–0.75) from internal validation, and 0.77 (95% CI = 0.74–0.80) from external validation using the Australian dataset. Both models were well calibrated in the external datasets (Hosmer-Lemeshow *P* = 0.57 for both).

## Discussion

Our comprehensive assessment of the contribution of common genomic variants to melanoma risk prediction showed that a polygenic risk score is strongly associated with melanoma risk and improved the classification of people at high risk of melanoma beyond that identified from traditional risk factors. The incremental improvement to risk prediction was independent of ambient sun exposure because the increases were similar for the Leeds and Australian studies.

The incremental AUCs (2.3% for Australia, 2.8% for Leeds) and NRIs (0.42 for Australia, 0.29 for Leeds), based on published risk estimates, indicate a moderate improvement to risk prediction. Similar improvements in the AUC have been shown when adding SNPs to an established breast cancer risk model (Howell et al., 2014), and smaller improvements were observed for prostate cancer (Szulkin et al., 2015) and colorectal cancer (Usher-Smith et al., 2016). Even relatively small improvements in the AUC have been shown to have clinical and public health utility and could be used in primary and secondary prevention to identify subgroups of the population at different levels of risk (Garcia-Closas et al., 2014). Genetic risk profiling is likely to contribute more in the future, because the proportion of variance in melanoma risk attributable to common genetic factors has been estimated to be at least 0.19 (Lu et al., 2014).

The observed discriminative ability of the polygenic risk score for melanoma was higher than has been previously reported. A Greek study observed a 1.1% increase in the AUC when adding information from 15 SNPs to a phenotypic risk model (Kypreou et al., 2016). A US study combining three different datasets found a statistically significant 3% increase in the AUC when adding 11 SNPs to a phenotypic risk model (Fang et al., 2013); however the NRI when based on categories of risk (<20%, 20%–50%, >50%) was not significant because improvements in sensitivity were outweighed by reduced specificity. Conversely, in our study we observed a significant NRI that was driven by improvements in specificity and sensitivity, particularly specificity. The Women’s Health Initiative cohort recently showed a 2-fold increased risk of melanoma for the highest versus lowest tertile of genetic risk score comprising 21 SNPs (Cho et al., 2018), compared with a 3-fold difference in our studies. They had limited information on phenotypic risk factors (Cho et al., 2018). We observed a larger improvement to risk prediction based on ORs from the study datasets, but this is likely due to model overfitting (Wray et al., 2013).

The similarities between the results from Australia and Leeds suggest that the published risk estimates used to create the polygenic risk score are appropriate across populations of European origin. Although the AUC values for the comprehensive model were different between countries (0.74 for Australia and 0.68 for Leeds), this simply reflects more pronounced differences in demographic factors (age, sex, ethnicity) between case and control individuals in Australia than in Leeds.

SNPs from genes in pigmentation pathways, particularly *MC1R*, contributed the most to risk prediction, despite several pigmentation phenotype variables already being included in the traditional risk factor model. *MC1R* influences risk through pigmentation and nonpigmentation pathways, and *MC1R* variants are associated with a higher relative risk for melanoma in people who do not have a high-risk pigmentation phenotype (Berwick et al., 2014, Cust et al., 2012, Kanetsky et al., 2010). This effect-measure modification of the association of *MC1R* with melanoma risk by phenotype (Pasquali et al., 2015) is the reason that we incorporated phenotype-stratified ORs for *MC1R* variants in the models. We previously showed a 2.1% increase in the AUC when adding *MC1R* genotype to a traditional risk model based on risk estimates derived from the Australian dataset (Cust et al., 2013), and a US study (Penn et al., 2014) observed a statistically significant 1% increase.

Despite nevi being one of the strongest risk factors for melanoma (Olsen et al., 2010a), SNPs from genes in nevus pathways did not improve risk prediction when based on published ORs, and they only modestly improved the AUC (by about 1%) when based on ORs derived from the dataset. Discrimination may improve with the discovery of new nevus-related genes (Duffy et al., 2017), but nevus phenotype measurement will likely continue to be important for risk prediction, perhaps because it reflects early-life sun exposure and genetic susceptibility (Bataille et al., 2000). SNPs from genes involved in other pathways unrelated to pigmentation and nevi, such as telomere length or senescence, were significantly associated with melanoma risk and made a modest contribution to risk prediction.

The more parsimonious risk prediction models that we developed showed good discrimination, because the AUCs remained at 0.72 or greater after internal and external validation. Not all SNPs and traditional risk factors were significantly associated with melanoma in the Australian and Leeds datasets, and the specific risk factors selected for the model differed between countries; this is probably a reflection of the studies’ sample sizes.

Although our cross-tabulation of polygenic with traditional risk score tertiles estimated that 59% of people in Australia and 49% in Leeds have a genetic risk concordant with their traditionally estimated risk, a considerable proportion (9% and 21%, respectively) had a high polygenic risk despite having a low traditional risk. These people might be the most likely to benefit from genomic profiling given that they do not have the visible risk factors identified in public health campaigns. Conversely, a similar proportion had a high traditional risk but a low polygenic risk; knowing they have a low genetic susceptibility might worsen their sun-related behaviors. Our stratified analyses suggest that the improvement in discrimination might be better for those with a low or average traditional risk score. Studies are underway to evaluate the impact on sun-related behaviors of giving personalized melanoma genomic risk information (Kanetsky and Hay, 2017, Smit et al., 2018).

There are several strengths of our analysis, including the population-based design, external validation in independent datasets using the same questionnaires, relatively large sample sizes, comprehensive risk factor information, and evaluation of SNPs in distinct biological pathways. We also account for known gene-environment interactions by using phenotype-stratified ORs for *MC1R* variants in the models. Overall interpretation of the lack of major differences between the two countries in our findings is restricted because of the differing age ranges at recruitment (younger than 40 years for Australia, no age limit for the UK). Thus, these results cannot necessarily be extrapolated to older Australians. Our two studies had a slightly different mix of European ethnicities, with more Eastern European ancestry in the Australian study. Although our results were similar when restricted to English ethnic background, these ethnic differences might be further reflected in different pigmentation phenotypes and sun-related behaviors. Further validation in larger, diverse datasets is warranted. We have underestimated the incremental contribution of the polygenic risk score because not all the functional variants were known, but this is likely a modest attenuation; conversely, *MC1R* functional variants were included, and this may have strengthened its contribution relative to other genes or pathways.

Combining common genomic predictors of melanoma risk with traditional melanoma risk factors improves the discrimination of melanoma risk prediction models and can identify as being at high risk an important fraction of people of European origin who are at high genetic risk but low traditional risk. Variants in *MC1R* were responsible for over half of the discrimination improvement, but SNPs in other genes further improved risk prediction. Prediction models based on both genomic and traditional risk factors could increase the yet unproven capacity of models based only on traditional risk factors to motivate melanoma risk reduction behaviors.

## Material and Methods

### Study samples

The Australian Melanoma Family Study was a multicenter, population-based, case-control family study of invasive cutaneous melanoma diagnosed between ages 18 and 39 years. Recruitment of case (n = 629) and control (n = 535) participants was locally coordinated in Sydney, Melbourne, and Brisbane, Australia. The study design, recruitment, data collection, and participant characteristics have been described (Cust et al., 2009), and more details for both studies are provided in the Supplementary Materials online.

The Leeds case-control study recruited population-based incident histopathologically confirmed invasive melanoma cases (n = 2,184) in patients aged between 18 and 82 years and population-ascertained control individuals (n = 513) (Newton-Bishop et al., 2011, Randerson-Moor et al., 2009). This analysis focuses on those case individuals whose measurement protocols exactly matched the Australian study (n = 964).

Approval for the study was obtained from the ethics committees of the coordinating centers’ institutions in Australia and Leeds and the cancer registries. All participants provided written informed consent.

### Self-reported personal sun exposure

Comprehensive data on lifetime sun exposure was collected by telephone interview. Questions referred to the frequency of sunburn and time spent outdoors between 9 a.m. and 5 p.m. separately for weekdays, weekends, and vacations in warmer months and in cooler months (Cust et al., 2011b, Newton-Bishop et al., 2011). Demographic information, ancestry, diagnoses of keratinocyte and other cancers, and family history information were also collected.

### Pigmentary and nevus phenotype

Participants reported the skin color of their inside upper arm, eye color, natural hair color at age 18 years, freckling using Gallagher’s freckle chart (Lee et al., 2005), ability to tan, propensity to sunburn, usual tanning and sunburn response to prolonged or repeated exposure of skin to sunlight in summer, and nevus density (described pictorially as none, few, some, many) (Cust et al., 2009).

### Selection of gene variants

We selected 21 genes/loci (45 SNPs) that had a confirmed association with melanoma risk in genome-wide association studies (Law et al., 2015) or for one gene using whole-genome sequencing approaches (*MITF* rs149617956 variant) (Yokoyama et al., 2011) (see Supplementary Table S3 online). Polygenic risk scores summarized the combined effects of the SNPs using a published method (Mavaddat et al., 2015) (for more technical details, see Supplementary Materials).

### Statistical analysis

#### Analytic dataset

We excluded participants who did not give a blood sample or who failed genotyping, were missing data on traditional risk factors for melanoma, had germline *CDKN2A* pathogenic mutations or non-European ethnicity (on self-report or principal components analysis), and Australian controls 45 years or older at interview (because all Australian cases were diagnosed when patients were younger than 40 years). The analysis dataset thus included 1,035 participants (578 case individuals, 457 control individuals) from Australia and 1,460 participants (964 case individuals, 496 control individuals) from Leeds.

#### Description of models

The primary analysis used ORs derived from published meta-analyses to prevent overfitting, that is, overestimation of the prediction accuracy that can occur when estimating the ORs from the same dataset that the predictions are made from (Steyerberg, 2009, Wray et al., 2013). The Australian and Leeds samples contributed data to the meta-analyses, but their data represented less than 10% of the total sample. In secondary analyses presented in Supplementary Table S2, we show the results based on ORs derived from the study datasets. The published ORs and ORs derived from the datasets are shown in Supplementary Table S3 for genomic variants and Supplementary Table S4 online for traditional risk factors. The published ORs for the traditional risk factors were from fully adjusted models in meta-analyses, and we categorized our variables the same as the published data. The published ORs for genomic variants were obtained from a meta-analysis of genome-wide association studies (Law et al., 2015), using pooled ORs from a fixed effects model or random effects where there was evidence of heterogeneity (*I*^2^ ≥ 31%). Because the association of *MC1R* with melanoma risk is modified by phenotype (Pasquali et al., 2015), we incorporated phenotype-stratified ORs for each of the *MC1R* variants for models that included traditional risk factors; for this stratification, participants were classified as having a sun-sensitive phenotype if they had one or more of freckles (few, some, many), red hair, or skin that usually or always burns.

Supplementary Table S3 shows the biological pathway/s through which each gene is thought to influence melanoma risk: pigmentation (14 genes); nevus (7 genes); and telomere, senescence, and other pathways (5 genes). Classification of pathways was based on associations of SNPs with each of these traits (Choi et al., 2017, Codd et al., 2013, Duffy et al., 2017, Law et al., 2015), and genes could be allocated to multiple pathways.

Logistic regression models were used to assess associations between melanoma and traditional and genomic risk factors, adjusted for demographic and study design factors: age, sex, city of recruitment (Australia only), and self-reported European ancestry (British, other Northern European, Southern/Eastern European, mixed/other European); ancestry was included as a covariate to minimize confounding by ethnicity. The base risk prediction model included well-established independent traditional risk factors and UV exposure variables (hair color, skin color, eye color, freckling as an adult, nevus density, reported skin photosensitivity, personal history of keratinocyte cancer, first-degree family history of melanoma, blistering sunburns as a child, vacation sun exposure, and sunbed use). The incremental contribution of melanoma risk SNPs was assessed overall, by biological pathway, and for *MC1R* alone.

#### Model performance

The ability of the model to discriminate between case and control individuals was evaluated by calculating the AUC, NRI, and OR per standard deviation (adjusted for age and sex using the OPERA method [Hopper, 2015]). The AUC is the probability that the predicted risk is higher for a case individual than for a control individual and ranges from 0.5 (equivalent to a coin toss) to 1.0 (perfect discrimination). The NRI quantifies overall improvement in model sensitivity and specificity; it quantifies movement of case and control individuals to higher or lower predicted risk probabilities when a new risk model is applied (Leening et al., 2014). Because there are no established risk thresholds for melanoma, we used category-free continuous NRI (Pencina et al., 2011). We used the Hosmer-Lemeshow goodness-of-fit test to assess calibration, that is, the agreement between observed and predicted probabilities of melanoma (Steyerberg, 2009). For the secondary analyses that used ORs derived from the study datasets, we performed 10-fold cross-validation as a measure of internal validation to help correct for overfitting (Steyerberg, 2009).

#### Cross-tabulation of polygenic risk score versus traditional risk score

Traditional and polygenic risk scores (each based on published risk estimates) were categorized into tertiles and cross-tabulated to compare the concordance of genomic with traditional risk. The traditional risk score variable was derived from the predicted probability of melanoma for each individual, based on β-values from a logistic regression model with melanoma (case-control) status as the outcome and traditional risk factors as predictors.

Data were analyzed using SAS, version 9.4 (SAS Institute, Cary NC), and statistical significance was inferred at two-sided *P* less than 0.05. We reported the study according to published guidelines (Collins et al., 2015, Janssens et al., 2011).

## ORCIDs

Anne E Cust: http://orcid.org/0000-0002-5331-6370

D Timothy Bishop: http://orcid.org/0000-0002-8752-8785

## Conflict of Interst

SM received grant funding from the Australian Research Council, Australian National Health and Medical Research Council. The other authors state no conflicts of interest.
