# Supplementary material for: Assessing the Incremental Contribution of Common Genomic Variants to Melanoma Risk Prediction in Two Population-Based Studies
Source: J Invest Dermatol. 2018 Dec;138(12):2617–24. doi: 10.1016/j.jid.2018.05.023 (PMC6249137; doi:10.1016/j.jid.2018.05.023)
Supplement: Supplementary Data [file mmc1.pdf]

Supplementary File Contents

SUPPLEMENTARY RESULTS 1
SUPPLEMENTARY METHODS 2
Study samples 2
Statistical fine mapping 3
Genotyping and imputation 4
Statistical analysis 4
SUPPLEMENTARY REFERENCES 5
SUPPLEMENTARY TABLES 8
Supplementary Table 1. Association of a pathway-specific polygenic risk score with melanoma risk in the Australian and Leeds studies 8
Supplementary Table 2: Incremental contribution of genetic risk factors to risk prediction of melanoma when added to a traditional risk factor model, based on risk estimates derived from the study datasets 11
Supplementary Table 3. Genomic variants included in the polygenic risk score, and their associations with melanoma and biological pathways 12
Supplementary Table 4. Traditional risk factors for melanoma included in the traditional risk factor base model 15

SUPPLEMENTARY RESULTS

Incremental contribution of polygenic risk score based on risk estimates derived from the study datasets

The incremental contribution of the polygenic risk score was stronger when the models were based on risk estimates derived from the study datasets (Supplementary Table 2). The polygenic risk score increased the AUC by 6.0% for Australia and 5.6% for Leeds, and by 2.0% and 1.2% respectively, when based on comparisons after 10-fold cross-validation. The NRI was 0.59 (95% CI 0.47, 0.71) for Australia and 0.48 (95% CI 0.37, 0.58) for Leeds; again this was driven more by improvements in specificity than sensitivity. Pigmentation-pathway SNPs, particularly in *MC1R*, again contributed the most improvement, but nevus pathway SNPs and telomere/senescence/other pathway SNPs also significantly improved net classification by 16% and 21% in Australia, respectively, and 22% and 17% in Leeds,

respectively, and each pathway modestly increased the AUC by about 1%. The Hosmer-Lemeshow tests indicated that each of the models was well calibrated.

Incremental contribution of polygenic scores, stratified by other factors

When we stratified by traditional risk tertiles, the incremental contribution of polygenic risk score appeared higher in the lowest tertile for traditional-risk for Leeds, and in the middle tertile for traditional-risk for Australia (data not shown). The incremental contribution of the polygenic risk score appeared lower for people with a family history (data not shown), although this was a small subgroup.

SUPPLEMENTARY METHODS

Study samples

In the Australian Melanoma Family Study, cases were identified from population-based state cancer registries, diagnosed between 1st July 2000 and 31st December 2002 with incident, histopathologically-confirmed, first-primary invasive cutaneous melanoma. Participation was 54% of those eligible and 76% of those contactable. Population controls were aged between 18 and 39 years at the time of approach and had no personal history of invasive or *in situ* melanoma. They were selected from the electoral roll (registration to vote is compulsory for Australian citizens aged 18 years and over) and were frequency-matched to cases by city, age (within 5 years) and sex. Participation of population controls was 23% of those eligible and 42% of those contactable. In addition, spouse/partner or friend controls were recruited through nomination by a case as a potential control subject. They were $\geq$ 18 years old and had

no history of invasive or *in situ* melanoma. A spouse or friend was nominated as a potential control subject by 59% of cases, and participation was 80% of those nominated.

In the Leeds case-control study, cases and controls were living in a geographically defined area of Yorkshire and the Northern region of the UK. Cases were identified through clinicians, pathology registers and the cancer registry (67% participation). Between September 2000 and June 2003, all people with invasive melanoma were invited to participate, and from July 2003 to September 2011, only cases with Breslow thickness ≥0.75mm were invited in order to enrich the cohort to observe clinical outcomes. Population-ascertained controls were identified by the cases' family doctors as not having cancer, and were randomly invited from individuals with the same sex and within the same 5-year age-group as a case. Controls had 55% participation. Overall 2,184 cases were recruited; however, this analysis excludes cases recruited in Leeds after 2007 who completed a shorter questionnaire which was distinct to the Australian questionnaire.

Statistical fine mapping

Statistical fine mapping of these loci was conducted jointly by the Leeds, Brisbane and Paris GenoMEL teams using data from 11,167 cases and 13,349 controls from the genome-wide meta-analysis (Law et al., 2015) to identify the set of distinct SNPs accounting for the melanoma association signals at each locus using a similar strategy (HyperLasso method) to that previously applied to the GenoMEL consortium data (Barrett et al., 2015). We identified 45 statistically independent (i.e. no or low linkage disequilibrium with other selected variants) single nucleotide polymorphisms (SNPs). Since the *MC1R* gene had been

previously sequenced, we included the sequenced genotype data instead of the finemapped SNPs for this gene (Cust et al., 2012).

Genotyping and imputation

Genotypes for the selected genetic variants were obtained for the Australian Melanoma Family Study and Leeds set through a combination of imputation, direct genotyping, and sequencing (*MC1R*). Australian samples were originally genotyped on Illumina Omni1-Quad arrays, and the Leeds samples on Illumina Human610-Quad array or the Illumina HumanOmniExpressExome BeadChip. Imputation was conducted genome-wide using IMPUTEv2.2 (Howie et al., 2009, Marchini and Howie, 2010). For quality control, we excluded SNPs with minor allele frequency <0.01, control Hardy-Weinberg Equilibrium P < 10^{-4}, or missingness >0.03. Samples were excluded if the call rate was <97%, there was evidence of non-European origin from principal components analysis, or evidence of first degree relationship with another sample. The 1000 Genomes April 2012 data (build 37) was the reference for imputation (Genomes Project et al., 2010). Only SNPs with a minor allele frequency >0.001 in the European reference panel were imputed. SNPs that were poorly imputed (quality score <0.8) were directly genotyped using the MassARRAY iPLEX® Gold assay for Australian samples and Taqman for Leeds samples.

Statistical analysis

Polygenic risk scores

Polygenic risk scores (overall and by biologic pathway) were derived as the sum of the minor alleles weighted by the per-allele log OR (Mavaddat et al., 2015), based on the assumption that SNP ORs combine multiplicatively (i.e. no interactions on a log-additive scale) as demonstrated previously (Mavaddat et al., 2015). *MC1R* variants were classified as 0 (none)

or 1 (any variant) rather than number of minor alleles (0,1,2), to be consistent with the published associations for *MC1R* stratified by phenotype (Pasquali et al., 2015).

Secondary analyses

Two *secondary analyses* were conducted. First, we included all the same risk factors in the models but derived ORs from the respective Australian and Leeds datasets rather than from published estimates. Second, instead of using all risk factors, we developed a more parsimonious risk prediction model using a backwards selection process in which traditional and genomic risk factors with $p < 0.20$ were retained in the multivariable model (Steyerberg, 2009). The same genetic variants and traditional risk factors were assessed for inclusion in both models. Study design features: age, sex, city of recruitment, and European ancestry, were also kept in the models.

SUPPLEMENTARY REFERENCES

Barrett JH, Taylor JC, Bright C, Harland M, Dunning AM, Akslen LA, et al. Fine mapping of genetic susceptibility loci for melanoma reveals a mixture of single variant and multiple variant regions. International journal of cancer 2015;136(6):1351-60.

Choi J, Xu M, Makowski MM, Zhang T, Law MH, Kovacs MA, et al. A common intronic variant of PARP1 confers melanoma risk and mediates melanocyte growth via regulation of MITF. Nat Genet 2017;49(9):1326-35.

Codd V, Nelson CP, Albrecht E, Mangino M, Deelen J, Buxton JL, et al. Identification of seven loci affecting mean telomere length and their association with disease. Nat Genet 2013;45(4):422-7, 7e1-2.

Colantonio S, Bracken MB, Beecker J. The association of indoor tanning and melanoma in adults: systematic review and meta-analysis. J Am Acad Dermatol 2014;70(5):847-57 e1-18.

Cust AE, Goumas C, Holland EA, Agha-Hamilton C, Aitken JF, Armstrong BK, et al. MC1R genotypes and risk of melanoma before age 40 years: A population-based case-control-family study. International journal of cancer 2012;131:E269–E81.

Dennis LK, Vanbeek MJ, Beane Freeman LE, Smith BJ, Dawson DV, Coughlin JA. Sunburns and risk of cutaneous melanoma: does age matter? A comprehensive meta-analysis. Ann Epidemiol 2008;18(8):614-27.

Duffy D, Zhu G, Li X, Sanna M, Iles M, Jacobs LC, et al. Novel pleiotropic risk loci for melanoma and nevus density implicate multiple biological pathways. bioRxiv 2017.

Gandini S, Sera F, Cattaruzza MS, Pasquini P, Picconi O, Boyle P, et al. Meta-analysis of risk factors for cutaneous melanoma: II. Sun exposure. Eur J Cancer 2005a;41(1):45-60.

Gandini S, Sera F, Cattaruzza MS, Pasquini P, Zanetti R, Masini C, et al. Meta-analysis of risk factors for cutaneous melanoma: III. Family history, actinic damage and phenotypic factors. Eur J Cancer 2005b;41(14):2040-59.

Genomes Project C, Abecasis GR, Altshuler D, Auton A, Brooks LD, Durbin RM, et al. A map of human genome variation from population-scale sequencing. Nature 2010;467(7319):1061-73.

Hopper JL. Odds per adjusted standard deviation: comparing strengths of associations for risk factors measured on different scales and across diseases and populations. Am J Epidemiol 2015;182(10):863-7.

Howie BN, Donnelly P, Marchini J. A flexible and accurate genotype imputation method for the next generation of genome-wide association studies. PLoS genetics 2009;5(6):e1000529.

Law MH, Bishop DT, Lee JE, Brossard M, Martin NG, Moses EK, et al. Genome-wide meta-analysis identifies five new susceptibility loci for cutaneous malignant melanoma. Nat Genet 2015;47(9):987-95.

Marchini J, Howie B. Genotype imputation for genome-wide association studies. Nat Rev Genet 2010;11(7):499-511.

Mavaddat N, Pharoah PD, Michailidou K, Tyrer J, Brook MN, Bolla MK, et al. Prediction of breast cancer risk based on profiling with common genetic variants. J Natl Cancer Inst 2015;107(5).

Olsen CM, Carroll HJ, Whiteman DC. Estimating the attributable fraction for melanoma: a meta-analysis of pigmentary characteristics and freckling. International journal of cancer 2010a;127(10):2430-45.

Olsen CM, Carroll HJ, Whiteman DC. Familial melanoma: a meta-analysis and estimates of attributable fraction. Cancer Epidemiol Biomarkers Prev 2010b;19(1):65-73.

Pasquali E, Garcia-Borron JC, Fargnoli MC, Gandini S, Maisonneuve P, Bagnardi V, et al. MC1R variants increased the risk of sporadic cutaneous melanoma in darker-pigmented Caucasians: a pooled-analysis from the M-SKIP project. International journal of cancer 2015;136(3):618-31.

Steyerberg EW. Clinical Prediction Models: A Practical Approach to Development, Validation, and Updating. Gail M, Tsiatis A, Krickeberg K, Wong M, Sarnet J, editors. New York, USA: Springer, 2009.

SUPPLEMENTARY TABLES

Supplementary Table 1. Association of a pathway-specific polygenic risk score with melanoma risk in the Australian and Leeds studies

	Australia				Leeds			
**Polygenic risk score by pathway**	**Range**	**Cases N (%)**	**Controls N (%)**	**Odds ratio[1] (95% CI)**	**Range**	**Cases N (%)**	**Controls N (%)**	**Odds ratio[1] (95% CI)**
**Pigmentation pathway**								
**Tertiles**								
Tertile 1	(-1.41 - 0.21)	101 (17)	146 (32)	1.00	(-1.06 - 0.21)	176 (18)	169 (34)	1.00
Tertile 2	(0.21 - 0.57)	158 (27)	170 (37)	1.33 (0.94, 1.89)	(0.21 - 0.57)	273 (28)	154 (31)	1.74 (1.30, 2.33)
Tertile 3	(0.57 - 2.36)	319 (55)	141 (31)	3.11 (2.21, 4.36)	(0.57 - 2.19)	515 (53)	173 (35)	2.94 (2.23, 3.88)
P trend				<0.0001				<0.0001
P int[2]								0.57
**Deciles**								
Decile 1)	(-1.41 - -0.16)	25 (4)	43 (9)	1.00	(-1.06 - -0.15)	37 (4)	53 (11)	1.00
Decile 2	(-0.15 - 0.05)	27 (5)	43 (9)	1.04 (0.51, 2.13)	(-0.15 - 0.05)	50 (5)	52 (10)	1.43 (0.80, 2.56)
Decile 3	(0.05 - 0.18)	40 (7)	52 (11)	1.35 (0.69, 2.64)	(0.05 - 0.18)	65 (7)	43 (9)	2.25 (1.26, 4.02)
Decile 4	(0.18 - 0.31)	43 (7)	38 (8)	1.97 (0.99, 3.91)	(0.18 - 0.31)	93 (10)	58 (12)	2.53 (1.47, 4.36)
Decile 5	(0.31 - 0.40)	60 (10)	84 (18)	1.22 (0.66, 2.28)	(0.31 - 0.40)	67 (7)	46 (9)	2.25 (1.27, 4.00)
Decile 6	(0.41 - 0.47)	27 (5)	19 (4)	2.50 (1.13, 5.56)	(0.41 - 0.48)	70 (7)	41 (8)	2.54 (1.42, 4.55)
Decile 7	(0.48 - 0.61)	54 (9)	51 (11)	1.63 (0.85, 3.12)	(0.48 - 0.61)	90 (9)	45 (9)	3.09 (1.76, 5.42)
Decile 8	(0.61 - 0.79)	74 (13)	42 (9)	2.91 (1.52, 5.57)	(0.61 - 0.79)	110 (11)	53 (11)	3.18 (1.84, 5.48)
Decile 9	(0.79 - 1.01)	73 (13)	41 (9)	2.73 (1.42, 5.23)	(0.79 - 1.01)	149 (15)	54 (11)	4.36 (2.56, 7.44)
Decile 10	(1.01 - 2.36)	155 (27)	44 (10)	5.75 (3.09, 10.72)	(1.01 - 2.19)	233 (24)	51 (10)	7.00 (4.12, 11.88)
P trend				<0.0001				<0.0001
P int[2]								0.76
OPERA[3]				1.71 (1.49, 1.96)				1.66 (1.49, 1.86)
P int[2]								0.76

Polygenic risk score by pathway	Australia Range	Australia Cases N (%)	Australia Controls N (%)	Australia Odds ratio[1] (95% CI)		Leeds Range	Leeds Cases N (%)	Leeds Controls N (%)	Leeds Odds ratio[1] (95% CI)	
**Nevus pathway**										
**Tertiles**										
Tertile 1	(-0.96 - -0.23)	164 (28)	141 (31)	1.00		(-1.01 - -0.23)	286 (30)	174 (35)	1.00	
Tertile 2	(-0.22 - -0.00)	211 (37)	179 (39)	1.03	(0.75, 1.40)	(-0.23 - -0.00)	313 (32)	145 (29)	1.30	(0.98, 1.71)
Tertile 3	(-0.00 - 0.75)	203 (35)	137 (30)	1.29	(0.93, 1.78)	(-0.00 - 0.92)	365 (38)	177 (36)	1.23	(0.95, 1.60)
P trend				0.12					0.13	
P int[2]									0.81	
**Deciles**										
Decile 1	(-0.96 - -0.47)	48 (8)	41 (9)	1.00		(-1.01 - -0.47)	68 (7)	55 (11)	1.00	
Decile 2	(-0.47 - -0.35)	51 (9)	49 (11)	0.95	(0.52, 1.72)	(-0.47 - -0.35)	89 (9)	49 (10)	1.60	(0.96, 2.64)
Decile 3	(-0.34 - -0.24)	48 (8)	42 (9)	1.14	(0.62, 2.11)	(-0.34 - -0.24)	99 (10)	50 (10)	1.67	(1.01, 2.74)
Decile 4	(-0.24 - -0.16)	58 (10)	40 (9)	1.42	(0.78, 2.60)	(-0.24 - -0.16)	100 (10)	56 (11)	1.48	(0.91, 2.40)
Decile 5	(-0.16 - -0.10)	107 (19)	102 (22)	1.01	(0.60, 1.70)	(-0.16 - -0.10)	86 (9)	33 (7)	2.13	(1.24, 3.66)
Decile 6	(-0.10 - -0.07)	24 (4)	18 (4)	1.36	(0.63, 2.93)	(-0.10 - -0.07)	83 (9)	37 (7)	1.82	(1.07, 3.10)
Decile 7	(-0.07 - 0.02)	61 (11)	38 (8)	1.44	(0.79, 2.64)	(-0.07 - 0.02)	108 (11)	59 (12)	1.54	(0.95, 2.49)
Decile 8	(0.02 - 0.13)	62 (11)	41 (9)	1.53	(0.84, 2.79)	(0.02 - 0.13)	119 (12)	53 (11)	1.85	(1.14, 3.00)
Decile 9	(0.13 - 0.29)	61 (11)	44 (10)	1.25	(0.69, 2.26)	(0.13 - 0.29)	109 (11)	51 (10)	1.75	(1.07, 2.85)
Decile 10	(0.29 - 0.75)	58 (10)	42 (9)	1.31	(0.72, 2.40)	(0.29 - 0.92)	103 (11)	53 (11)	1.64	(1.00, 2.68)
P trend				0.10					0.10	
P int[2]									0.85	
OPERA[3]				1.09 (0.96, 1.24)					1.10 (0.98, 1.23)	
P int[2]									0.96	

Polygenic risk score by pathway	Australia Range	Australia Cases N (%)	Australia Controls N (%)	Australia Odds ratio[1] (95% CI)	Leeds Range	Leeds Cases N (%)	Leeds Controls N (%)	Leeds Odds ratio[1] (95% CI)
**Telomere, senescence, other pathway**								
**Tertiles**								
Tertile 1	(-0.35 - 0.11)	149 (26)	143 (31)		(-0.40 - 0.11)	267 (28)	172 (35)	1.00
Tertile 2	(0.12 - 0.25)	207 (36)	173 (38)	1.09 (0.79, 1.50)	(0.12 - 0.25)	281 (29)	151 (30)	1.23 (0.93, 1.62)
Tertile 3	(0.25 - 0.75)	222 (38)	141 (31)	1.40 (1.01, 1.93)	(0.25 - 0.82)	416 (43)	173 (35)	1.55 (1.19, 2.02)
P trend				0.04				0.001
P int[2]								0.79
**Deciles**								
Decile 1	(-0.35 - -0.05)	40 (7)	43 (9)		(-0.40 - -0.05)	83 (9)	53 (11)	1.00
Decile 2	(-0.05 - 0.03)	43 (7)	42 (9)	1.03 (0.55, 1.93)	(-0.05 - 0.03)	66 (7)	53 (11)	0.79 (0.48, 1.31)
Decile 3	(0.03 - 0.10)	52 (9)	42 (9)	1.33 (0.72, 2.46)	(0.03 - 0.10)	83 (9)	53 (11)	1.00 (0.61, 1.64)
Decile 4	(0.10 - 0.15)	59 (10)	46 (10)	1.17 (0.63, 2.14)	(0.10 - 0.15)	112 (12)	50 (10)	1.50 (0.92, 2.43)
Decile 5	(0.15 - 0.18)	89 (15)	97 (21)	0.97 (0.56, 1.67)	(0.15 - 0.18)	62 (6)	30 (6)	1.29 (0.74, 2.27)
Decile 6	(0.18 - 0.21)	30 (5)	24 (5)	1.17 (0.57, 2.41)	(0.18 - 0.21)	88 (9)	41 (8)	1.37 (0.82, 2.28)
Decile 7	(0.21 - 0.27)	64 (11)	32 (7)	2.02 (1.07, 3.79)	(0.21 - 0.27)	95 (10)	63 (13)	0.99 (0.62, 1.59)
Decile 8	(0.27 - 0.34)	69 (12)	44 (10)	1.43 (0.79, 2.60)	(0.27 - 0.34)	125 (13)	50 (10)	1.61 (0.99, 2.59)
Decile 9	(0.34 - 0.42)	60 (10)	41 (9)	1.47 (0.80, 2.71)	(0.34 - 0.42)	100 (10)	55 (11)	1.15 (0.71, 1.86)
Decile 10	(0.42 - 0.75)	72 (12)	46 (10)	1.52 (0.84, 2.74)	(0.42 - 0.82)	150 (16)	48 (10)	2.00 (1.24, 3.23)
P trend				0.03				0.001
P int[2]								0.92
OPERA[3]				1.15 (1.01, 1.31)				1.18 (1.06, 1.31)
P int[2]								0.76

[1] Models are adjusted for demographic and study design factors: age, sex, city of recruitment and European ancestry.
[2] P-value for interaction comparing trends across countries
[3] OR per adjusted standard deviation, stratified by country (Australia/Leeds) and adjusted for age and sex, using the OPERA method (Hopper, 2015)

Supplementary Table 2: Incremental contribution of genetic risk factors to risk prediction of melanoma when added to a traditional risk factor model, based on risk estimates derived from the study datasets

Risk factor model	AUC (95% CI)	Change in AUC from base model	P-value[1]	Hosmer-Lemeshow P-value	Improvement in sensitivity NRI (95% CI)[2]	Improvement in specificity NRI (95% CI)[2]	Overall improvement in classification NRI (95% CI)[2]
**Australia (N=1,035)**							
Base model with traditional risk factors[3]	0.75 (0.72, 0.78)			0.47			
+ *MC1R*	0.78 (0.75, 0.81)	0.027	0.0003	0.70	0.11 (0.03, 0.19)	0.26 (0.17, 0.34)	0.37 (0.25, 0.49)
+ Pigmentation pathway	0.80 (0.77, 0.83)	0.049	<0.0001	0.11	0.22 (0.14, 0.30)	0.33 (0.24, 0.42)	0.55 (0.43, 0.67)
+ Nevus pathway	0.76 (0.73, 0.79)	0.007	0.08	0.64	-0.07 (-0.15, 0.01)	0.23 (0.14, 0.32)	0.16 (0.04, 0.28)
+ Telomere, senescence & other pathway	0.76 (0.74, 0.79)	0.012	0.006	0.19	0.07 (-0.01, 0.15)	0.14 (0.05, 0.23)	0.21 (0.09, 0.33)
+ All SNPs[4]	0.81 (0.79, 0.84)	0.060	<0.0001	0.14	0.26 (0.18, 0.34)	0.33 (0.24, 0.41)	0.59 (0.47, 0.71)
**Leeds (N=1,460)**							
Base model with traditional risk factors[3]	0.69 (0.66, 0.72)			0.84			
+ *MC1R*	0.72 (0.69, 0.75)	0.029	0.0002	0.89	-0.04 (-0.10, 0.03)	0.40 (0.32, 0.48)	0.36 (0.26, 0.47)
+ Pigmentation pathway	0.74 (0.71, 0.76)	0.048	<0.0001	0.44	0.17 (0.11, 0.23)	0.25 (0.16, 0.33)	0.42 (0.31, 0.52)
+ Nevus pathway	0.70 (0.68, 0.73)	0.013	0.016	0.46	0.09 (0.03, 0.15)	0.13 (0.04, 0.22)	0.22 (0.11, 0.33)
+ Telomere, senescence & other pathway	0.69 (0.67, 0.72)	0.005	0.19	0.73	0.00 (-0.06, 0.07)	0.17 (0.08, 0.26)	0.17 (0.07, 0.28)
+ All SNPs[4]	0.75 (0.72, 0.77)	0.056	<0.0001	0.86	0.21 (0.14, 0.27)	0.27 (0.19, 0.35)	0.48 (0.37, 0.58)

AUC Area under the receiver operating characteristic curve, NRI Net reclassification improvement.

[1] Chi-square p-value for the difference in the AUC when compared to the base model.

[2] Based on continuous NRI. Improvement in sensitivity is calculated from reclassification of cases, improvement in specificity is calculated from reclassification of controls, and the overall improvement combines the improvements in sensitivity and specificity.

[3] Traditional factors include hair colour, skin colour, eye colour, freckling as an adult, skin photosensitivity, self-reported nevi, sunbed use, keratinocyte cancer personal history, first degree family history of melanoma, holiday sun exposure and blistering sunburns as a child; as well as demographic and study design factors: age, sex, city of recruitment and European ancestry.

[4] Added as a polygenic risk score, comprising 45 SNPs in 21 genes. The SNPs in each pathway can overlap; the pigmentation pathway includes 14 genes (31 SNPs), nevus includes 7 genes (13 SNPs), and telomere, senescence, and other pathways includes 5 genes (9 SNPs). The polygenic risk score increased the AUC by 2.0% for Australia and 1.2% for Leeds when based on comparisons after 10-fold cross-validation.

Supplementary Table 3. Genomic variants included in the polygenic risk score, and their associations with melanoma and biological pathways

									Pathway[e]		
**Region/ Gene**	**Chromosome**	**Single nucleotide polymorphisms (SNP)**	**Minor allele**	**Major allele**	**Minor allele frequency[a]**	**Published Odds Ratio (95% CI)[b]**	**Australia dataset Odds Ratio (95% CI)[c]**	**Leeds dataset Odds Ratio (95% CI)[d]**	**Pigmentation**	**Nevus**	**Telomere, Senescence, other**
PARP1	1	rs3219090	T	C	0.328	0.88 (0.84, 0.92)	0.77 (0.62, 0.94)	0.95 (0.80, 1.12)			X
ARNT	1	rs7412746	C	T	0.453	0.89 (0.85, 0.92)	0.82 (0.67, 0.99)	0.84 (0.72, 0.99)	X		
CYP1B1/ RMDN2	2	rs1056837	A	G	0.440	1.09 (1.03, 1.14)[f]	0.97 (0.79, 1.18)	1.06 (0.91, 1.24)	X	X	
CASP8	2	rs700635	C	A	0.272	1.11 (1.07, 1.15)	0.88 (0.70, 1.09)	1.25 (1.05, 1.50)			X
MITF	3	rs149617956	A	G	0.007	1.54 (1.27, 1.82)	1.32 (0.38, 4.55)	1.61 (0.46, 5.71)	X	X	
TERT	5	rs6554679	T	C	0.227	1.08 (1.04, 1.12)	1.06 (0.85, 1.33)	1.09 (0.90, 1.31)			X
		rs2736100	A	C	0.504	0.93 (0.87, 0.98)[f]	0.81 (0.67, 0.99)	1.00 (0.86, 1.18)			X
		rs36115365	C	G	0.203	1.04 (0.99, 1.08)	0.92 (0.72, 1.17)	0.99 (0.81, 1.20)			X
		rs466502	G	A	0.441	1.16 (1.13, 1.20)	1.08 (0.89, 1.31)	1.09 (0.93, 1.27)			X
		rs2550948	C	T	0.398	1.05 (1.01, 1.09)	1.00 (0.82, 1.22)	1.06 (0.90, 1.26)			X
SLC45A2	5	rs16891982	C	G	0.046	0.50 (0.24, 0.76)	0.49 (0.25, 0.94)	0.88 (0.51, 1.52)	X		
CDKAL1	6	rs7776158	A	G	0.316	1.11 (1.07, 1.15)	1.17 (0.96, 1.43)	1.29 (1.08, 1.53)	X		
		rs12527588	C	T	0.053	1.23 (1.16, 1.31)	1.42 (0.91, 2.21)	0.95 (0.66, 1.37)	X		
AGR3	7	rs34585474	T	C	0.112	1.14 (1.09, 1.20)	1.00 (0.75, 1.35)	1.04 (0.81, 1.32)	X		
		rs73069846	T	C	0.396	1.11 (1.07, 1.15)	1.11 (0.91, 1.37)	1.09 (0.93, 1.28)	X		
		rs7781130	C	T	0.045	1.22 (1.13, 1.30)	0.89 (0.55, 1.46)	1.49 (0.98, 2.28)	X		
		rs6949072	C	A	0.138	1.10 (1.04, 1.16)	1.36 (1.04, 1.77)	0.95 (0.75, 1.19)	X		
CDKN2A	9	rs871024	A	C	0.514	0.83 (0.77, 0.88)[f]	0.92 (0.75, 1.13)	1.17 (0.99, 1.37)		X	
		rs3731217	C	A	0.143	0.86 (0.81, 0.92)	0.83 (0.62, 1.10)	0.77 (0.61, 0.96)		X	
		rs1011970	T	G	0.171	1.14 (1.09, 1.20)[f]	1.29 (0.98, 1.68)	0.95 (0.78, 1.15)		X	
		rs77560034	C	G	0.097	1.18 (1.11, 1.24)	1.11 (0.79, 1.55)	0.96 (0.74, 1.24)		X	
RAD23B	9	rs1484375	A	G	0.227	1.12 (1.08, 1.16)	1.07 (0.85, 1.35)	0.99 (0.82, 1.20)	X	X	
		rs4436178	A	G	0.041	1.18 (1.07, 1.29)[f]	1.77 (1.07, 2.94)	1.64 (1.06, 2.54)	X	X	
		rs113908778	T	C	0.031	1.28 (1.18, 1.37)	1.18 (0.70, 2.02)	1.04 (0.67, 1.61)	X	X	
OBFC1	10	rs2487999	T	C	0.100	1.14 (1.08, 1.19)	1.36 (0.97, 1.91)	1.26 (0.95, 1.67)			X
TYR	11	rs1393350	A	G	0.269	1.22 (1.18, 1.26)[f]	1.29 (1.04, 1.59)	1.37 (1.15, 1.63)	X		

										Pathway[e]		
**Region/ Gene**	**Chromosome**	**Single nucleotide polymorphisms (SNP)**	**Minor allele**	**Major allele**	**Minor allele frequency[a]**	**Published Odds Ratio (95% CI)[b]**	**Australia dataset Odds Ratio (95% CI)[c]**	**Leeds dataset Odds Ratio (95% CI)[d]**	**Pigmentation**	**Nevus**	**Telomere, Senescence, other**
CCND1	11	rs9651783	G	T	0.348	1.12 (1.09, 1.16)	0.98 (0.80, 1.19)	1.08 (0.92, 1.28)	X	X	
		rs76699054	A	G	0.095	0.86 (0.78, 0.94)[f]	1.19 (0.84, 1.70)	0.80 (0.61, 1.04)	X	X	
ATM	11	rs1801516	A	G	0.142	0.84 (0.79, 0.89)	0.96 (0.72, 1.27)	0.90 (0.72, 1.14)		X	
OCA2	15	rs4778138	G	A	0.139	0.84 (0.78, 0.90)	0.89 (0.67, 1.19)	0.86 (0.67, 1.10)	X		
FTO	16	rs16953002	A	G	0.159	1.15 (1.10, 1.20)	0.98 (0.75, 1.26)	1.18 (0.95, 1.47)			X
MC1R (not stratified by phenotype)											
	16	rs1805005 (V60L)	T	G	0.123	1.00 (0.92, 1.07)	1.28 (0.94, 1.74)	0.87 (0.69, 1.09)	X		
		rs1805006 (D84E)	A	C	0.015	1.46 (1.26, 1.67)	1.53 (0.79, 2.96)	0.86 (0.49, 1.51)	X		
		rs2228479 (V92M)	A	G	0.092	1.12 (1.06, 1.18)	1.19 (0.85, 1.65)	0.99 (0.76, 1.27)	X		
		rs11547464 (R142H)	A	G	0.008	1.47 (1.06, 2.03)	1.57 (0.58, 4.24)	1.04 (0.41, 2.61)	X		
		rs1805007 (R151C)	T	C	0.102	1.85 (1.79, 1.91)	2.44 (1.78, 3.36)	2.11 (1.61, 2.76)	X		
		rs1805008 (R160W)	T	C	0.094	1.37 (1.31, 1.43)	1.18 (0.86, 1.63)	1.46 (1.13, 1.90)	X		
		rs885479 (R163Q)	A	G	0.047	1.04 (0.95, 1.12)	0.93 (0.61, 1.44)	0.70 (0.48, 1.02)	X		
		rs1805009 (D294H)	C	G	0.021	1.89 (1.57, 2.28)	1.13 (0.68, 1.87)	1.92 (1.07, 3.45)	X		
		rs1110400 (I155T)	T	G	0.012	1.30 (1.07, 1.52)[f]	2.40 (1.12, 5.13)	1.46 (0.70, 3.08)	X		
MC1R – Sun-sensitive phenotype[g]											
	16	rs1805005 (V60L)				0.95	1.25 (0.79, 1.99)	0.90 (0.64, 1.25)			
		rs1805006 (D84E)				1.56	1.49 (0.66, 3.38)	1.05 (0.52, 2.14)			
		rs2228479 (V92M)				1.04	1.48 (0.86, 2.54)	0.97 (0.67, 1.39)			
		rs11547464 (R142H)				1.84	0.99 (0.33, 2.95)	0.83 (0.27, 2.52)			
		rs1805007 (R151C)				1.77	2.72 (1.77, 4.17)	2.20 (1.57, 3.08)			
		rs1805008 (R160W)				1.27	1.48 (0.94, 2.34)	1.84 (1.27, 2.67)			
		rs885479 (R163Q)				1.56	1.18 (0.55, 2.51)	0.79 (0.47, 1.33)			
		rs1805009 (D294H)				1.61	2.65 (1.17, 6.02)	3.08 (1.37, 6.97)			
		rs1110400 (I155T)				0.76	4.15 (1.13, 15.18)	1.27 (0.52, 3.09)			
MC1R – Sun-resistant phenotype[g]											
		rs1805005 (V60L)				2.51	1.42 (0.93, 2.19)	0.69 (0.49, 0.97)			
		rs1805006 (D84E)				4.13	1.74 (0.52, 5.81)	0.59 (0.24, 1.47)			
		rs2228479 (V92M)				3.13	1.08 (0.68, 1.71)	0.87 (0.60, 1.26)			

									Pathway[e]		
**Region/ Gene**	**Chromosome**	**Single nucleotide polymorphisms (SNP)**	**Minor allele**	**Major allele**	**Minor allele frequency[a]**	**Published Odds Ratio (95% CI)[b]**	**Australia dataset Odds Ratio (95% CI)[c]**	**Leeds dataset Odds Ratio (95% CI)[d]**	**Pigmentation**	**Nevus**	**Telomere, Senescence, other**
		rs11547464 (R142H)				3.75	Unavailable	1.59 (0.31, 8.00)			
		rs1805007 (R151C)				5.55	2.06 (1.25, 3.38)	1.53 (0.99, 2.36)			
		rs1805008 (R160W)				6.69	1.04 (0.64, 1.70)	1.64 (0.45, 5.93)			
		rs885479 (R163Q)				2.6	0.97 (0.55, 1.69)	0.87 (0.58, 1.31)			
		rs1805009 (D294H)				14.36	0.62 (0.28, 1.40)	0.54 (0.30, 0.97)			
		rs1110400 (I155T)				1.83	1.77 (0.67, 4.63)	0.87 (0.36, 2.10)			
ASIP	20	rs62211989	C	G	0.085	1.43 (1.35, 1.50)[f]	1.74 (1.27, 2.40)	1.47 (1.12, 1.95)	X		
		rs7274597	T	C	0.069	0.87 (0.81, 0.94)	0.55 (0.37, 0.84)	0.90 (0.66, 1.23)	X		
MX2	21	rs6517661	C	A	0.112	0.91 (0.85, 0.97)	1.01 (0.74, 1.38)	0.85 (0.67, 1.08)	X		
		rs45430	C	T	0.395	0.87 (0.83, 0.90)	0.80 (0.65, 0.98)	0.89 (0.75, 1.04)	X		
PLA2G6	22	rs132985	T	C	0.473	0.89 (0.85, 0.94)[f]	1.14 (0.94, 1.38)	0.82 (0.70, 0.96)	X	X	

[a] Minor allele frequency in controls, calculated as a weighted average from the control samples in the individual datasets in the genome-wide meta-analysis (Law et al., 2015)

[b] Unless otherwise indicated, per-allele odds ratios for melanoma and 95% confidence intervals were obtained from a meta-analysis of genome-wide association studies (Law et al., 2015), using the pooled odds ratio from a fixed effects model. Odds ratios for R142H and D294H were obtained from the online Melgene database meta-analysis due to ≤ 2 studies with data available in the meta-analysis dataset.

[c] Per-allele odds ratios for melanoma derived from the Australian Melanoma Family Study dataset.

[d] Per-allele odds ratios for melanoma derived from the Leeds case-control study dataset.

[e] Classification of pathways was based on associations of SNPs with phenotype characteristics (Choi et al., 2017, Codd et al., 2013, Duffy et al., 2017, Law et al., 2015), and each gene could be allocated to more than 1 pathway.

[f] Odds ratios were from a random effects model in these cases where the I^2 was ≥ 31%, indicating the presence of heterogeneity.

[g] As the association of *MC1R* variants with melanoma risk is modified by phenotype, we incorporated phenotype-stratified odds ratios for each of the *MC1R* variants for models that also included traditional risk factors, according to whether participants had one or more of: freckles (few, some, many), red hair, or skin that usually or always burns (Pasquali et al., 2015). Phenotype-stratified odds ratios were analysed as 0 (none) versus 1 (any variant) to be consistent with Pasquali et al.

Supplementary Table 4. Traditional risk factors for melanoma included in the traditional risk factor base model

Variable[1]	Published Odds Ratio (95% CI)	Reference	Australia dataset[2]	Leeds dataset[3]
Family history of melanoma				
None	1.00	(Olsen et al., 2010b)	1.00	1.00
1 or more	2.05 (1.68,2.50)		1.74 (1.16, 2.62)	2.97 (1.47, 6.00)
Non-melanoma skin cancer				
No	1.00	(Gandini et al., 2005b)	1.00	1.00
Yes	4.28 (2.80, 6.55)		2.64 (1.42, 4.91)	1.67 (0.80, 3.48)
Skin colour				
Olive or Brown	1.00	(Gandini et al., 2005b)	1.00	1.00
Fair	2.06 (1.68, 2.52)		1.13 (0.71, 1.79)	1.15 (0.77, 1.71)
Freckles as an adult				
None	1.00	(Olsen et al., 2010a)	1.00	1.00
Few/Some/Many	1.91 (1.64, 2.21)		0.95 (0.69, 1.29)	0.97 (0.75, 1.25)
Skin phototype				
Never/Sometimes burns	1.00	(Olsen et al., 2010a)	1.00	1.00
Usually/Always burns	1.99 (1.51, 2.63)		1.17 (0.86, 1.60)	1.41 (1.08, 1.85)
Eye colour				
Brown	1.00	(Olsen et al., 2010a)	1.00	1.00
Green or Hazel	1.43 (1.19, 1.71)		0.90 (0.60, 1.36)	0.85 (0.60, 1.20)
Blue or Grey	1.49 (1.28, 1.74)		0.83 (0.54, 1.25)	1.09 (0.77, 1.53)
Hair colour				
Dark Brown or Black	1.00	(Olsen et al., 2010a)	1.00	1.00
Light Brown	1.28 (1.07, 1.53)		1.13 (0.80, 1.59)	1.39 (1.04, 1.85)
Fair or Blonde	1.73 (1.42, 2.12)		2.23 (1.44, 3.47)	1.75 (1.24, 2.48)
Red	2.10 (1.77, 2.49)		4.36 (2.30, 8.23)	2.76 (1.73, 4.41)
Intermittent sun exposure[4]				
Lowest quartile	1.00	(Gandini et al., 2005a)	1.00	1.00
Highest 3 quartiles	1.61 (1.31, 1.99)		0.83 (0.60, 1.16)	0.82 (0.61, 1.09)
Childhood blistering sunburns				
None	1.00	(Dennis et al., 2008)	1.00	1.00
One or more	1.71 (1.40, 2.09)		0.91 (0.67, 1.23)	0.96 (0.63, 1.45)
Sunbed use				
0 sessions	1.00	(Colantonio et al., 2014)	1.00	1.00
1-10 sessions	1.07 (0.90, 1.26)		1.01 (0.65, 1.55)	0.87 (0.63, 1.19)
>10 sessions	1.34 (1.05, 1.71)		1.80 (1.04, 3.10)	0.88 (0.66, 1.18)
Self-reported nevus density				
None	1.00	Combined study datasets (unpublished)	1.00	1.00
Few	1.61 (1.24, 2.09)		1.08 (0.56, 2.06)	1.80 (1.27, 2.55)
Some	3.35 (2.52, 4.44)		2.62 (1.37, 4.99)	3.62 (2.45, 5.33)
Many	5.11 (3.61, 7.24)		4.23 (2.09, 8.56)	4.59 (2.77, 7.62)

[1] Data were self-reported.

[2] Odds ratios derived from the Australian Melanoma Family Study dataset, adjusted for all other variables in the table.

[3] Odds ratios derived from the Leeds case-control study dataset, adjusted for all other variables in the table.

[4] Holiday/vacation sun exposure was used for 'intermittent' exposure for the Australian and Leeds datasets.
